# Durable complete response to neoantigen-loaded dendritic-cell vaccine following anti-PD-1 therapy in metastatic gastric cancer

**DOI:** 10.1038/s41698-022-00279-3

**Published:** 2022-06-03

**Authors:** Zengqing Guo, Yuan Yuan, Chao Chen, Jing Lin, Qiwang Ma, Geng Liu, Yan Gao, Ying Huang, Ling Chen, Li-Zhu Chen, Yu-Fang Huang, Hailun Wang, Bo Li, Yu Chen, Xi Zhang

**Affiliations:** 1 Department of Medical Oncology, Fujian Medical University Cancer Hospital & Fujian Cancer Hospital, Fuzhou, Fujian Province China; 2 Cancer Bio-immunotherapy Center, Fujian Medical University Cancer Hospital & Fujian Cancer Hospital, Fuzhou, Fujian Province China; 3 Fujian Provincial Key Laboratory of Translational Cancer Medicine, Fuzhou, Fujian Province China; 4 BGI-Shenzhen, Shenzhen, 518083 China; 5 College of Life Sciences, University of Chinese Academy of Sciences, Beijing, 100049 China

**Keywords:** Gastric cancer, Cancer immunotherapy, Drug development, Immunotherapy, Translational research

## Abstract

Neoantigens are ideal targets for dendritic cell (DC) vaccines. So far, only a few neoantigen-based DC vaccines have been investigated in clinical trials. Here, we reported a case of a patient with metastatic gastric cancer who received personalized neoantigen-loaded monocyte-derived dendritic cell (Neo-MoDC) vaccines followed by combination therapy of the Neo-MoDC and immune checkpoint inhibitor (ICI). The patient developed T cell responses against neoantigens after receiving the Neo-MoDC vaccine alone. The following combination therapy triggered a stronger immune response and mediated complete regression of all tumors for over 25 months till October, 2021. Peripheral blood mononuclear cells recognized seven of the eight vaccine neoantigens. And the frequency of neoantigen-specific T cell clones increased obviously after vaccination. Overall, this report describing a complete tumor regression in a gastric cancer patient mediated by Neo-MoDC vaccine in combination with ICI, and suggesting a promising treatment for patients with metastatic gastric cancer.

## Introduction

Dendritic cells (DCs) are specialized antigen-presenting cells and play important roles in antitumor immunity. It has been reported that cross-presentation by DCs was required for effective anti-PD-1 (Programmed cell death protein 1) treatment^1^. The antitumor effects of PD-1 antibody were abolished in DC-deficient mouse^2^. Furthermore, increased number of DCs within tumor milieu has been reported to be associated with increased overall survival^3^ or responsiveness to anti-PD-1 immunotherapy^4^ in cancer patients.

Tumor-associated antigens (TAAs) loaded DC vaccines in cancer treatment have been extensively investigated in clinical trials^5,6^. The overall response rate to these DC vaccines was about 8–15% in clinical trials in patients with melanoma, prostate cancer, glioblastoma, or renal cell carcinoma^7,8^. Although TMB (tumor mutation burden) does not always correlate with ICI (immune checkpoint inhibitor) response^9,10^, neoantigen load displayed strong correlation with responsiveness to ICI treatment^11,12^, therefore neoantigens are considered as ideal targets for DC vaccines. Neoantigen-based DC vaccines were used in clinical trials treating melanoma^13^, and pancreatic adenocarcinoma^14^, and proposed to treat ovarian cancer patients^15^. Only three melanoma patients’ clinical results were reported to date. So far, neoantigen-based DC vaccines has not been tested in treating gastric cancer patients in clinical trials.

Here, we reported a patient with advanced metastatic gastric cancer who was treated with Neo-MoDC (personalized neoantigen-loaded monocyte-derived dendritic cell) vaccine and ICI for the first time. The Neo-MoDC vaccines were administrated alone in the first two months, followed by combination therapy with nivolumab. The Neo-MoDC vaccines induced neoantigen-specific CD4^+^ and CD8^+^ T cell activation, and increased the frequency of neoantigen-specific T cell clones obviously in peripheral blood. Tumor volume decreased rapidly after the initiation of the combination treatment. And multiple doses of Neo-MoDC-ICI induced complete regression for 25 months till now (October 2021). To our best knowledge, it is the first study of achieving complete and durable gastric tumor regression by neoantigen-based DC vaccine and ICI therapy.

## Results

### Neo-MoDC vaccine generation

A 66-year-old female patient was diagnosed with gastric cancer (stage IV, pT4aN2M1, Bormann type III) in July 2017. Then laparoscopic-assisted D2 radical distal gastrectomy was conducted, and multiplex IHC of primary tumor tissue showed low PD-1 expression but high PD-L1 expression (20% of staining cells; Supplementary Fig. 1). FOLFOX treatment was performed. Eight months later, metastases were found in her peritoneal and lymph nodes. After two cycles of Nab-paclitaxel administration, the patient could not bear the treatment and quitted her chemotherapy.

The patient was then enrolled in our ongoing clinical trial (NCT03185429). Whole exome sequencing of primary tumor tissues and peripheral blood revealed 1,096 somatic non-synonymous mutations (Supplementary Table 1). The HLA type of this patient was HLA-A02:07, HLA-A24:02, HLA-B40:01, HLA-B 46:01, HLA-C01:02, and HLA-C03:04. According to predicted binding affinity of these mutant peptides with the patient’s HLA class I alleles, eight soluble mutant epitopes with highest affinity were identified, and each corresponding 27mer mutant peptides were synthesized (Supplementary Table 2). Upon clonal architecture analysis, these eight mutant epitopes were distributed in subclones 1 or 2 in the Kernel density plots (Supplementary Fig. 2a).

Next, Neo-MoDC vaccines were generated in cGMP (current Good Manufacturing Practices) grade manufacturing facilities. Autologous dendritic cells were generated from monocytes in PBMCs. And these monocytes-derived dendritic cells were loaded with peptides and then activated with cytokine cocktail. We then assessed DC maturation by measuring the surface expression of HLA-DR, CD80, CD83, CD86, and CD14, and found that Neo-MoDC cells expressed high level of HLA-DR, CD80, CD83, CD86, and decreased level of CD14 (Supplementary Fig. 2b, c). Meanwhile, cytokine secretion assay also showed increased production of IL23 after activation of mature DC (Supplementary Fig. 2d). These results confirmed that the autologous Neo-MoDC cells generated were mature and functioning.

### Combination therapy mediated durable complete tumor regression

**Fig. 1:**
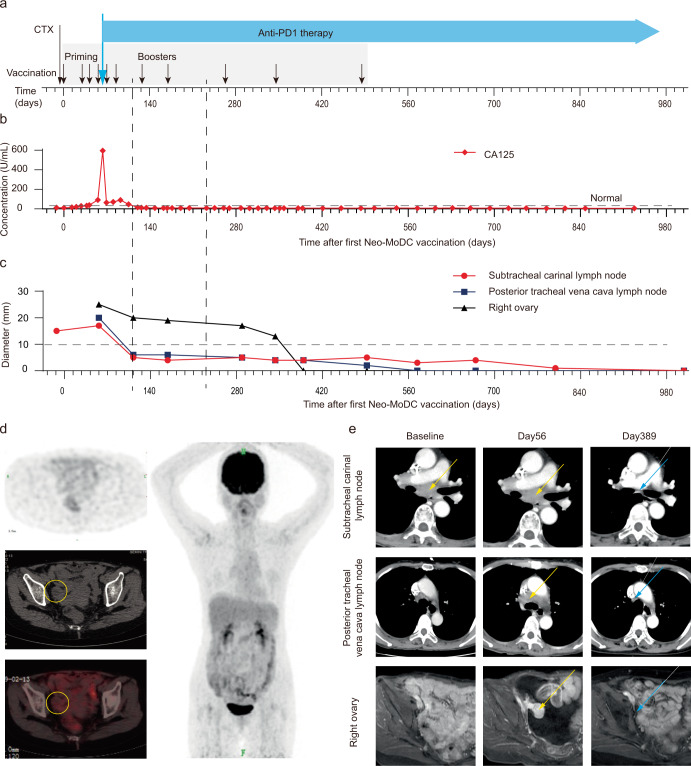
The combination immunotherapy of Neo-MoDC vaccine and nivolumab mediated rapid complete regression. **a** Treatment schema. Cyclophosphamide (CTX) 300 mg/m^2^ was administrated two days before the first dose of Neo-MoDC vaccine. **b** The changes of tumor marker CA125 during the treatment. **c** Tumor lesions shrank during treatment and the follow-ups. Two vertical dotted lines denote day 126 (lymph node < 10 mm) and day 231 (tumor complete regression by PET/CT). **d** PET/CT image of right ovary (Left panels, yellow circles) and PET image (right panel) show complete regression on day 231. **e** Representative images of targeted tumor lesions during treatment. Yellow arrows represent targeted tumor lesions. Blue arrows denote complete regressed tumor lesions.

**Fig. 2:**
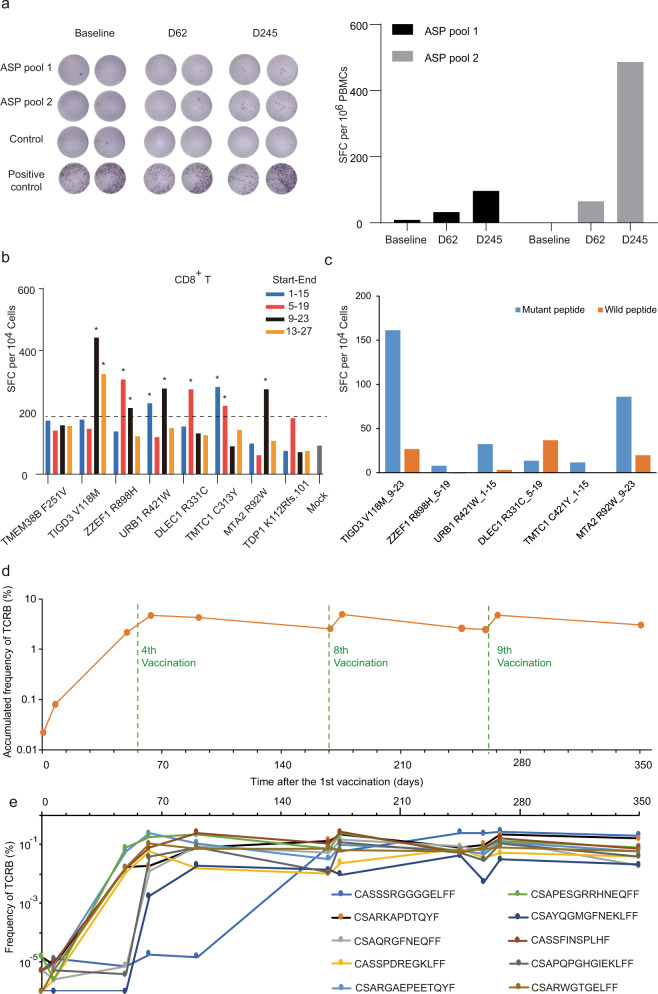
Neoantigen-specific immunological monitoring. **a** Scans of ELISPOT (left panel) and quantification (right panel) results showed day62 PBLs (after DC vaccination, before ICI treatment) and day245 PBLs (after the combination treatment) generated more IFN-γ spots than day-6 PBLs (before vaccination) when induced by ASP pools 1 or 2. ASP pool 1, 15mer assay mutant peptides pool of *TMEM38B* F251V, *TIGD3* V118M, *ZZEF1* R898H, and *URB1* R421W; ASP pool 2, 15mer assay mutant peptide pool of *DLEC1* R331C, *TMTC1* C313Y, *MTA2* R92W, and *TDP1* K112Rfs.101. The positive control for ELISPOT was PMA/ionomycin at the concentration of 1 ng/mL PMA and 500 ng/mL ionomycin. The control is media only. **b** Mapping of CD8^+^ T cells response to each 15mer peptide. Each 27mer mutant peptide was cutting into four 15mer peptides (denoted by blue, red, black, and orange bars) whose start and end positions in the 27mer peptide are 1-to-15, 5-to-19, 9-to-23, and 13-to-27 (also shown in Supplementary Fig. 3). Dotted line, 2 folds of mock (media only); *, mutant epitope with SFC number > 2 folds than that of mock. **c** ELISPOT assays showed that 5 out of 6 tested mutant peptides, but not their corresponding WT peptides, specifically activated CD8^+^ T cells. Data shown here were the mean value of two replicates. **d** Total frequency of neoantigen-specific TCRB clones increased during vaccination. **e** Neoantig**e**n-specific TCRB clones, whose frequencies were in top 10 in tumor tissue, changed in PBLs during vaccination.

Before DC vaccination, the patient underwent lymphodepletion with cyclophosphamide (300 mg/m^2^). Then Neo-MoDC vaccines were administrated subcutaneously alone to prime T cells to elicit antitumor immune responses in the first two months (Fig. 1a). When we examined the T cell activity in the peripheral blood using ELISPOT assay, we saw that the Neo-MoDC vaccination induced neoantigen-specific T cell responses in this patient (Day62, Fig. 2a). However, tumor still progressed in this patient in these two months.

Then Nivolumab was given every 14 days since day 65. The initiation of combination treatment with nivolumab and Neo-MoDC vaccines led to a rapid regression of all tumor lesions. We examined the serum CA-125 (cancer antigen 125) level which is a biomarker of gastric cancer progression^16,17^. Five days after the first round of Nivolumab treatment (day 65 in Fig. 1a), serum CA-125 level decreased rapidly from 596 to 64 U/ml (Fig. 1b). Two weeks later, patient’s malignant ascites disappeared, and the volume of metastatic supraclavicular lymph nodes metastases shrank by ~30% based on clinical imaging examination. Two months later, the short axis of all the metastatic lymph nodes reduced to < 10 mm, and patients achieved complete response (CR) in these lymph nodes according to RECIST guideline^18^. Meanwhile, the long diameter of ovarian implantation metastasis reduced by 20% (Fig. 1c).

On day 231 after the initial vaccination, tumor burden in the patient was assessed by PET scan. And the imaging indicated a complete regression of all lesions (Fig. 1d). The CT scan on day 389 during the combination therapy showed that the ovarian implantation metastasis also disappeared completely (Fig. 1e). Re-staging CT showed the complete regression remains for 25 months till now (October, 2021).

### Neo-MoDC vaccination-induced neoantigen-specific T cell responses

To study T cell response to Neo-MoDC vaccination, we collected peripheral blood lymphocytes (PBLs) at several time points during vaccination. We first constructed 15mer mutant assay peptide (ASP) pools from 27mer mutant epitopes (Supplementary Fig. 3), and explored immunogenicity to ASP pools by ex vivo interferon–γ (IFN–γ) ELISPOT assay by using PBLs from day-6 (before vaccination) and on day62 (after the vaccination, before ICI treatment). We found that the day62 PBLs generated more IFN-γ obviously than that of day-6 PBLs when induced by ASP pools 1 or 2 (Fig. 2a). Furthermore, day37 and day62’s PBLs expressed more CD137 obviously than that of PBLs before Neo-MoDC vaccination (Supplementary Fig. 4). These results demonstrated that Neo-MoDC vaccination-induced neoantigen-specific T cell responses before nivolumab injection. Next, we studied the neoantigen-specific T cell responses after the combination treatment with the Neo-MoDC vaccination and ICI. Results showed that the number of IFN-γ spots generated from day245 PBLs were 3 folds (ASP pool 1) and 7 folds (ASP pool 2) more than those from day62 PBLs (Fig. 2a), which demonstrated that a much more potent effect of combination treatment in inducing neoantigen-specific T cells responses. Furthermore, our data also showed that mutant peptides activated T cells and upregulated the level of PD-1 (Supplementary Fig. 4), which further suggest that the PD-1/PD-L1 axis could be one reason that neo-DC alone was not effective in this patient.

To identify which mutant epitopes mediate T cell responses, we incubated PBLs (day79) with pooled 15mer mutant epitopes, cultured them for 21 days, and tested the immunogenicity of individual 15mer mutant epitopes. Our results showed that activated CD8^+^ T could recognize 15mer mutant epitope from 6 of the 8 vaccine peptides including *TIGD3* V118M, *URB1* R421W, *ZZEF1* R898H, *DLEC1* R331C, *TMTC1* C313Y, and *MTA2* R92W (Fig. 2b). And ELISPOT assays showed that 5 out of 6 tested mutant peptides, but not their corresponding WT peptides, specifically activated CD8^+^ T cells (Fig. 2c). Although our algorithm predicts only HLA class I binding for CD8^+^ cells, surprisingly, activated CD4^+^ T cells recognized 7 of the 8 vaccine peptides (except *ZZEF1* R898H) (Supplementary Fig. 5a). And 2 out of the 5 tested mutant peptides, but not their corresponding WT peptides, specifically activated CD4^+^ T cells (Supplementary Fig. 5b). Among these peptides, two 15mer mutant epitopes (9-23 and 13-27) of *TIGD3* V118M can activate both CD8^+^ and CD4^+^ T cell responses.

### Frequency of neoantigen-specific T cell clones increased during treatment

To further study the dynamics of T cell activation during vaccination, we collected primary tumor tissue, blood samples at different time points before and during vaccination, activated PBLs (CD107A^+^) with mutant peptides, sequenced CDR3-regions of TCRB chain, and analyzed TCRB clonotypes. We compared TCRBs in the tumor tissue and blood, and counted the number of overlapped TCRB clones. Results showed that 35.3%-86.5% of TCRB clones in PBLs could be found in tumor tissue at different PBL sampling time points (Supplementary Fig. 6a). We then focused on those tumor-enriched TCRB clones which have a frequency in primary tumor tissue 10-fold higher than that in day0 PBLs. Results showed that the frequency of those enriched TCRB clones in PBLs increased obviously (from 0% to 16.8%) after four doses of DC vaccine, and the frequency stayed relatively stable during the following vaccinations (Supplementary Fig. 6b, c).

Next, we activated T cells (CD107A^+^) in the PBLs in vitro with mutant peptides and analyzed TCRB clonotypes. We found that 88.8% of CD107A^+^ TCRB clones in PBLs were presented in TCRB repertoire of tumor tissue (Supplementary Fig. 6d), suggesting that most of activated T cells (CD107A^+^) in PBLs also existed in tumors and could have similar anti-tumor function to T cells in tumor tissue. We then defined these TCRB clones as neoantigen-specific TCRB clones if they can be found in both tumor tissues and in mutant peptide-activated PBLs. A total of 217 neoantigen-specific TCRB clones were identified.

To study the effect of vaccination on neoantigen-specific TCRB clones, we analyzed the frequency of these neoantigen-specific TCRB clones during the treatment. Result showed that the accumulated frequency of all these clones increased rapidly from 0.022% to 2.18% in PBLs after four doses of Neo-MoDC vaccine. The accumulated frequency stayed relative stable after the fourth dose of vaccines, but increased temporarily within two weeks after the eighth and the ninth dose of vaccines (Fig. 2d). Next, we selected top 10 neoantigen-specific TCRB by their frequency in tumor tissue, and studied their frequency in PBLs during vaccination. Most of these TCRB clones displayed similar pattern in frequency alteration after vaccination (Fig. 2e). In addition, we examined high-frequency T cell clones with a frequency higher than 0.1%, 0.05%, or 0.01% in PBLs, and found that the number of T cell clones in all these three frequencies increased rapidly from the first to the fourth vaccination (Supplementary Fig. 7). Similarly, the number of high-frequency clones increased temporarily within two weeks after the eighth and the ninth dose of vaccines.

## Discussion

ICI therapy has been tested in gastric cancer patients in clinic recently. Several phase I/Ib studies showed some efficacy of the immune checkpoint inhibitors pembrolizumab and nivolumab in chemotherapy-refractory gastric cancer, with ORRs between 14-23% and CR rates between 0-2% (Checkmate-032, KEYNOTE-028, KEYNOTE-012)^19,20^. However, several phase III trials in advanced gastric cancer either failed to show a survival benefit (Pembrolizumab) or minimal survival benefit (ATTRACTION-4, CheckMate 649)^21–23^. Therefore, new types of immunotherapy such as the combination therapy with DC vaccine are urgently needed.

In our current study, the administration of Neo-MoDC vaccine alone in the first two months was able to induce neoantigen-specific T cell responses and increase the frequency of neoantigen-specific T cell clones in this patient. However, tumor still progressed in this patient in these two months. One possible explanation is the immunosuppressive tumor microenvironment, such as the expression of PD-L1 in the tumor. ICI treatment altered the immunosuppressive tumor microenvironment, and enhanced neoantigen-specific T cells responses (Fig. 2a). The patient showed rapid and significant tumor regression after adding ICI. Interestingly, our data showed that Neo-MoDC vaccine activated T cells and upregulated the level of PD-1 (Supplementary Fig. 4). These results suggest that Neo-MoDC and ICI may enhance the efficacy of each other in combination therapy, and support using a combination treatment of neo-DC with ICI in the future. Data collected from more patients will help to further illustrate the contribution of neo-DC vs ICI during DC vaccine treatment.

There is a controversy that if clonal neoantigens are more important in immunotherapy than subclonal neoantigens. In previous studies, clonal neoantigens were reported to be enriched in non-small cell lung cancer and melanoma patients who were sensitive to immune checkpoint inhibitor (ICI) therapy^24^. Clonal neoantigen load was associated with overall survival in patients treated with ICI therapy^25^. Clonal neoantigen such as *KRAS* G12D^26^ would be an ideal target in neoantigen-based therapy. However, clonal neoantigen copy-number loss was also reported^27^ in cancer patients, and Marty et al. reported that clonal neoantigens were often poorly presented by tumor cells^28^, which all contribute to tumor immune escape. On the other side, Luksza et al.’s study indicated that tumors having high-quality neoantigens in multiple subclones were equal to having good clonal neoantigens^29^. In our study, using a cutoff of 0.75 to differentiate truncal from subclonal mutations with SCHISM^30,31^, we found that most of the mutations (1043/1096) were subclonal, and all the predicted candidate neoantigens with strong binding affinities were derived from subclonal mutations. Furthermore, 5 of all the 8 vaccine subclonal neoantigens can mediate both CD4^+^ and CD8^+^ T cell responses, indicating that these subclonal neoantigens are high-quality and can be used in neoantigen-based therapy.

Neoantigen-based DC vaccine is considered to be one main type of immunotherapy in the future, and being rigorously tested in many clinical trials worldwide. However, accurate identification of immunogenic neoantigens is challenging. Currently, most neoantigen prediction algorithms based mainly on binding affinity or presentation data to find HLA-matched neoantigens, and cannot provide information about which peptides can induce T cell activation. Therefore, the average rate of success in identifying immunogenic neoantigens is far below our expectation. Meanwhile, validation of predicted neoantigens, large-scale DC cell maturation and vaccine production usually will take 1-3 months to complete. Timing is critical for advanced cancer patients, therefore advanced algorithms and easier and faster-to-produce neoantigen-based DC vaccines are needed before it can be routinely used to treat cancer patients.

Neoantigen-based DC vaccines was able to broaden the diversity of neoantigen-specific T cells and induce 30% tumor reduction for three months in one melanoma patient^13^. Neoantigen-based DC vaccines were reported to start testing in pancreatic adenocarcinoma^14^ and ovarian cancer^15^ patients in 2019. However, results of these two clinical trials have not been published to date. In our current study, the combined therapy of Neo-MoDC vaccine and ICI mediated completely tumor regression in an advanced metastatic gastric cancer patient from our ongoing clinical trial. To our best knowledge, this is the first study of achieving complete and durable tumor regression by neoantigen-based DC vaccine and ICI therapy.

In conclusion, we demonstrated the Neo-MoDC vaccination in conjunction with ICI mediated a complete regression of metastatic gastric cancer, which is still ongoing for > 25 months now. The vaccination induced neoantigen-specific CD4^+^ and CD8^+^ T cell activation, and increased the frequency of neoantigen-specific T cell clones. These neoantigen immunogenicity most likely contributed to the complete response of the patient. This study suggests a novel combination immunotherapy approach for treating metastatic gastric cancers.

## Methods

### Study design and ethics approval

The ongoing clinical investigations (NCT03185429) is an open-labeled, single-arm phase I study in metastatic gastrointestinal cancer patients. The protocol was approved by Ethics Committee of Fujian Cancer Hospital, and the patient provided written informed consent before enrollment. Primary endpoints were safety, while secondary endpoints were efficacy. In the safety assessment, adverse events were categorized and graded according to Common Terminology Criteria for Adverse Events (CTCAE) Version 5.0. Clinical efficacy assessment was according to Response Evaluation Criteria in Solid Tumors (RECIST) 1.1.

### Neoantigen prediction

DNA was extracted from primary tumor lesion in the stomach (formalin fixed paraffin-embedded) and peripheral blood. Whole-exome capture libraries were constructed, and whole-exome sequencing was performed on Hiseq4000 platform. After filtering with SOAPnuke^32^, high-quality reads were mapped to hg19 genome with Edico (http://evonexus.org/evonexus-companies/edico-genome/). Reads, mapped around insertions and deletions (indels) region, were re-mapping locally by using GATK. Single nucleotide variations (SNVs) and indels were calling through using Mutect^33^ and Strelka^34^, respectively.

Somatic mutations were annotated by ANNOVAR^35^. 8- to 11-mer mutant peptides were generated. Binding affinity were predicted by using five programs (NetMHC^36^, NetMHCpan^37^, PSSMHCpan^38^, PickPocket^39^, and SMM^40^), and a comprehensive affinity score was calculated. If there are no more than two values with IC50 < 500, the affinity score will be 50000 (i.e. no affinity). If there are three or more values with IC50s < 500, its final affinity score will be the smallest value out of the five IC50s. Mutant peptides will stay if they meet the following criteria: 1) mutation were supported by more than five reads; 2) IC50 of SNV-derived mutant peptides were smaller than 20; 3) IC50 of indel-derived mutant peptides were smaller than 50.

### Neo-MoDC generation and administration

Eight long peptides (purity>98%, Genscript) were synthesized for DC vaccine generation. Each peptide was 27-mer with the selected mutated amino acid at position 14. Peptides were dissolved and filtered using 0.22μm syringe filter. Autologous peripheral blood mononuclear cells (PBMCs) were isolated from leukapheresis sample using Ficoll paque (GE Healthcare Life Sciences) density gradient centrifugation. Autologous CD14^+^ monocytes were isolated from peripheral blood mononuclear cells (PBMCs) using CliniMACS CD14 reagent (Miltenyi Biotec), and cultured in T175 culture flasks in X-VIVO 15 medium (LONZA) supplemented with GM-CSF (1000U/ml, Gibco), IL-4 (Life technology), and 2% human serum albumin (CSL BEHRING). Three days later, immatured DCs (imDCs) were pulsed with 10 ug/ml synthesized peptides overnight. These imDCs were matured with cytokine cocktails containing TNF-α (10 ng/ml, PEPROTECH), PGE2 (1ug/ml, SIGMA), IL-1b (10 ng/ml, PEPROTECH), and IL-6 (1000 U/ml, PEPROTECH) for 40 h. The mature Neo-MoDCs were washed twice in saline and cryopreserved in liquid nitrogen (around 1×10^7^ cells/ml).

Phenotype of DCs were analyzed by flow cytometry using a panel of markers: CD83-APC (BD Biosciences, clone: HB15e, 551073), CD80-PE (Biolegend, clone: 2D10, 305208), CD86-FITC (Biolegend, clone: BU63, 374204), CD14-APC (Biolengend, clone:M5E2, 301808), HLA-DR-PE (eBioscience, clone: L243, 12-9952-42). The use of each antibody was 5 µl per million cells in 100 µl staining volume.

The lymphodepletion treatement with cyclophosphamide (300 mg/m^2^ daily) were performed two days before the first dose of vaccine. Neo-MoDC vaccines (2.0×10^7^−4.0×10^7^ DCs per dose) were administered subcutaneously on day 0, 30, 42, 56, 70, 85, 127 and intranodally on day 169, 262, 344 and 483 (Fig. 1a). The combination treatment with Nivolumab (240 mg intravenously every 2 weeks) was started on day 65. No adjuvants were used in the Neo-MoDC vaccination. Average cell viability was around 90% post-thaw.

### Neoantigen-specific immune response detection

Ex vivo IFN-γ ELISPOT assays were conducted to monitor neoantigen-specific immune response. Cryopreserved PBMCs were thawed, resuspended at a density of 2×10^6^ cells per ml in RPMI1640 medium (GIBCO) supplemented with 10% fetal bovine serum (FBS) (Hyclone), and rested overnight in 37°C 5% CO_2_ incubator. 2-3×10^5^ PBMCs per well were stimulated using assay peptides (ASP) pools for 16-20 h. A neoantigen-specific response was reported if the number of spot-forming cells (per 10^6^ PBMC) was >55, and > 2-fold than that of control wells.

To determine the immunogenicity and epitope of each long peptides, in vitro expansion of neoantigen-specific T cells was performed on PBMC isolated from leukapheresis sample collected at day79. Briefly, 2×10^6^ PBMCs were stimulated in 48-well cell culture plates with pooled ASP peptides (each at 2 μg/ml) in X-VIVO 15 medium (LONZA) supplemented with 20 IU/mL IL-2 and 10% FBS. Half medium change and supplementation of peptides and IL-2 were performed at 3-day intervals. After10-21 days, CD8^+^ or CD4^+^ T cells were isolated using CD8^+^ or CD4^+^ T cell MicroBeads (Miltenyi Biotec) for IFN-γ ELISPOT assay. 5×10^3^ (Figs. 2b) or 2×10^4^ (Fig. 2c) CD8^+^ T cells or 2-3×10^4^ CD4^+^ cells were co-cultured with autologous DCs pulsed with individual peptide (2 μg/ml) at ratio T:DC = 5:1.

### TCR sequencing and analysis

The primary tumor lesion in stomach (surgically removed, formalin fixed and paraffin-embedded (FFPE)) was used for TCRB sequencing and establishing “neoantigen-T cells clones”. First, the tumor DNA was isolated from FFPE samples using a QIAamp DNA FFPE Tissue Kit. Then the third complementary determinant region (CDR3) of TCR beta-chains was amplified by multiplex PCR and sequenced using the method described previously^41^. Briefly, 32 forward primers annealed to the FR3 region, and 13 reverse primers annealed to the junction (J) region of TCR were used (Supplementary Table 3). The amplified product was purified by electrophoresis on 2% agarose gel, and sequenced on DNB-seq platform (paired-end 100). The modified IMonitor software^42^ was used for basic statistics and clonotype identification. Briefly, Fastp software^43^ was used to filter out low-quality reads, and then pair-end reads were assembled and aligned to IMGT database (IMGT, http://www.imgt.org/) for identification of TCR sequences.

### Reporting summary

Further information on research design is available in the Nature Research Reporting Summary linked to this article.

## Supplementary information

Supplementary material

REPORTING SUMMARY

## Data Availability

Sequencing data are deposited in CNGB Nucleotide Sequence Archive (CNSA: https://db.cngb.org/cnsa; accession number CNP0000871), as approved by the Ethics Committee of Fujian Cancer Hospital. Readers may also contact the corresponding authors for access to individual patient-level data for non-commercial purposes only.

## Code availability

SOAPnuke2.0.5, Strelka2, NetMHC-3.0, NetMHCpan-3.0, and R (version 4.0.2) were used for data analysis. No custom code or algorithms were created.
