# Supplementary material for: Durable complete response to neoantigen-loaded dendritic-cell vaccine following anti-PD-1 therapy in metastatic gastric cancer
Source: NPJ Precis Oncol. 2022 Jun 3;6:34. doi: 10.1038/s41698-022-00279-3 (PMC9166775; doi:10.1038/s41698-022-00279-3)
Supplement: Supplementary file 1 — Supplementary material [file 41698_2022_279_MOESM1_ESM.pdf]

# Supplementary Material

**Durable complete response to neoantigen-loaded dendritic-cell vaccine following anti-PD-1 therapy in metastatic gastric cancer**

Zengqing Guo[1,3,4,*], Yuan Yuan[2,5,*], Chao Chen[2,*], Jing Lin[1,3,4,*], Qiwang Ma[2,*], Geng Liu[2], Yan Gao[2], Ying Huang[2], Ling Chen[1,3,4], Li-Zhu Chen[1,3,4], Yu-Fang Huang[1,3,4], Hailun Wang[2], Bo Li[2,†], Yu Chen[1,3,4,†], Xi Zhang[2,†]

† *Correspondence to*: xzresearch@163.com (X. Z.); chenyu1980@fjmu.edu.cn (Y.C.); libo@genomics.cn (B.L.);

**This file includes:**

Supplementary Tables 1 to 3

Supplementary Figures 1 to 7

**Supplementary tables**

Supplementary Table 1. Sequencing data summary

| Tumor | | | Normal | | | Somatic nonsynonymous mutations |
|---|---|---|---|---|---|---|
| Raw data (Gb) | Depth (×) | 10× coverage (%) | Raw data (Gb) | Depth (×) | 10× coverage (%) | |
| 163.76 | 242.02 | 99.62 | 114.06 | 208.43 | 99.72 | 1,096 |

Supplementary Table 2. Mutant epitope for Neo-MoDC vaccine

| **ID** | **Name** | **Peptide sequences** |
|---|---|---|
| 1 | TMEM38B F251V | DTLSWMLFGWQQPVSSCEKKSEAKSPS |
| 2 | TIGD3 V118M | KAKELADIMGQDFMPSIGWLVRWKRRN |
| 3 | ZZEF1 R898H | TEQEHKQSLQLTFHSLCTYFSDKDPGG |
| 4 | URB1 R421W | SRAFQTREFIPLPWLLAMVMVTTVPLV |
| 6 | DLEC1 R331C | ARMESRNHFLKNPCFFPPNTRYGGKSL |
| 7 | TMTC1 C313Y | VAERVLYMPSMGYYILFVHGLSKLCTW |
| 9 | MTA2 R92W | QRHQLKHRELFLSWQFESLPATHIRGK |
| 11 | TDP1 K112Rfs.101 | TLEPSTSRIFYLLYLGRLFLQLSLTTA |

Supplementary Table 3. List of the TRB primers used in this study, including primer names and sequences.

| **TRB V Primers (32 forward primers)** | |
|---|---|
| TRBV2 | ATTTCACTCTGAAGATCCGGTCCAC |
| TRBV3-1 | AAACAGTTCCAAATCGMTTCTCAC |
| TRBV4-1/2/3 | CAAGTCGCTTCTCACCTGAATG |
| TRBV5-1 | GCCAGTTCTCTAACTCTCGCTCT |
| TRBV5-4/5/6/8 | TCAGGTCGCCAGTTCCCTAAYTAT |
| TRBV6-4.1 | CACGTTGGCGTCTGCTGTACCCT |
| TRBV6-8/5/1.2 | CAGGCTGGTGTCGGCTGCTCCCT |
| TRBV6-9/7/1.1/6 | CAGGCTGGAGTCAGCTGCTCCCT |
| TRBV6-4.2 | AGTCGCTTGCTGTACCCTCTCAG |
| TRRBV6-2/3 | GGGGTTGGAGTCGGCTGCTCCCT |
| TRBV7-2/4/6/7/8 | GGGATCCGTCTCCACTCTGAMGAT |
| TRBV7-3 | GGGATCCGTCTCTACTCTGAAGAT |
| TRBV7-9 | GGGATCTTTCTCCACCTTGGAGAT |
| TRBV9 | CCTGACTTGCACTCTGAACTAAACCT |
| TRBV10-1 | CCTCACTCTGGAGTCTGCTGCC |
| TRBV10-2/3 | CCTCACTCTGGAGTCMGCTACC |
| TRBV11-1/2/3 | GCAGAGAGGCTCAAAGGAGTAGACT |
| TRBV12-3.2/5.2 | GAAGGTGCAGCCTGCAGAACCCAG |
| TRBV12-3.1/4/5.1 | GAAGATCCAGCCCTCAGAACCCAG |
| TRBV13 | TCGATTCTCAGCTCAACAGTTC |
| TRBV14 | GGAGGGACGTATTCTACTCTGAAGG |
| TRBV15 | TTCTTGACATCCGCTCACCAGG |
| TRBV16 | CTGTAGCCTTGAGATCCAGGCTACGA |
| TRBV18 | TAGATGAGTCAGGAATGCCAAAG |
| TRBV19 | TCCTTTCCTCTCACTGTGACATCGG |
| TRBV20-1 | AACCATGCAAGCCTGACCTT |
| TRBV24-1 | CTCCCTGTCCCTAGAGTCTGCCAT |
| TRBV25-1 | GCCCTCACATACCTCTCAGTACCTC |
| TRBV27-1 | GATCCTGGAGTCGCCCAGC |
| TRBV28 | ATTCTGGAGTCCGCCAGC |

| | |
|---|---|
| TRBV29-1 | AACTCTGACTGTGAGCAACATGAG |
| TRBV30-F5 | CAGATCAGCTCTGAGGTGCCCCA |

| **TRB J Primers (13 reverse primers)** | |
|---|---|
| TRBJ1.1 | CTTACCTACAACTGTGAGTCTGGTG |
| TRBJ1.2 | CTTACCTACAACGGTTAACCTGGTC |
| TRBJ1.3 | CTTACCTACAACAGTGAGCCAACTT |
| TRBJ1.4 | AAGACAGAGAGCTGGGTTCCACT |
| TRBJ1.5 | CTTACCTAGGATGGAGAGTCGAGTC |
| TRBJ1.6 | CATACCTGTCACAGTGAGCCTG |
| TRBJ2.1 | CCTTCTTACCTAGCACGGTGA |
| TRBJ2.2 | CTTACCCAGTACGGTCAGCCT |
| TRBJ2.3 | CCGCTTACCGAGCACTGTCAG |
| TRBJ2.4 | AGCACTGAGAGCCGGGTCC |
| TRBJ2.5 | CGAGCACCAGGAGCCGCGT |
| TRBJ2.6 | CTCGCCCAGCACGGTCAGCCT |
| TRBJ2.7 | CTTACCTGTGACCGTGAGCCTG |

Note:

These primers are from PMID: 26297338.

**Supplementary figures**

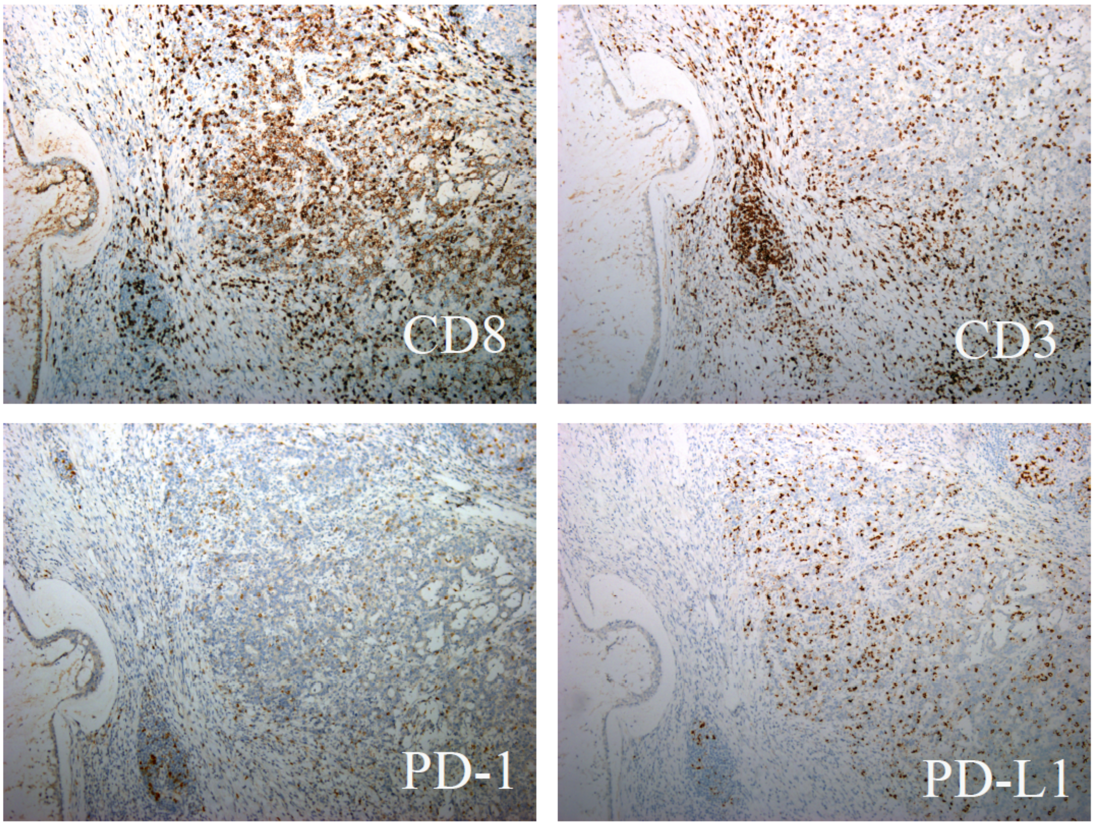


**Supplementary Figure 1. The staining of CD8, CD3, PD-1, and PD-L1 on resected primary gastric tumor tissue.** The results showed that there were many CD8 T cells in the tumor tissue. PD-1 expression level was low in the tumor, but PD-L1 showed strong staining. And 20% of tumor cells and immune cells were PD-L1 positive in the tumor tissue. Immunohistochemistry for CD3, CD8, PD-L1, PD-1 were performed with CONFIRM anti-CD3 (2GV6) antibody (VENTANA, 790-4341), (790-4460) CONFIRM CD8 (SP57) antibody (VENTANA, 790-4460), PD-L1 (SP142) Assay (VENTANA, 740-4859) and anti-PD-1 antibody (MXB Biotechnologies, MAB-0743) using an automated slide stainer (BenchMark XT, VENTANA Medical Systems, Tucson, AZ, USA). The antibodies were directly added into the stainer without dilution.

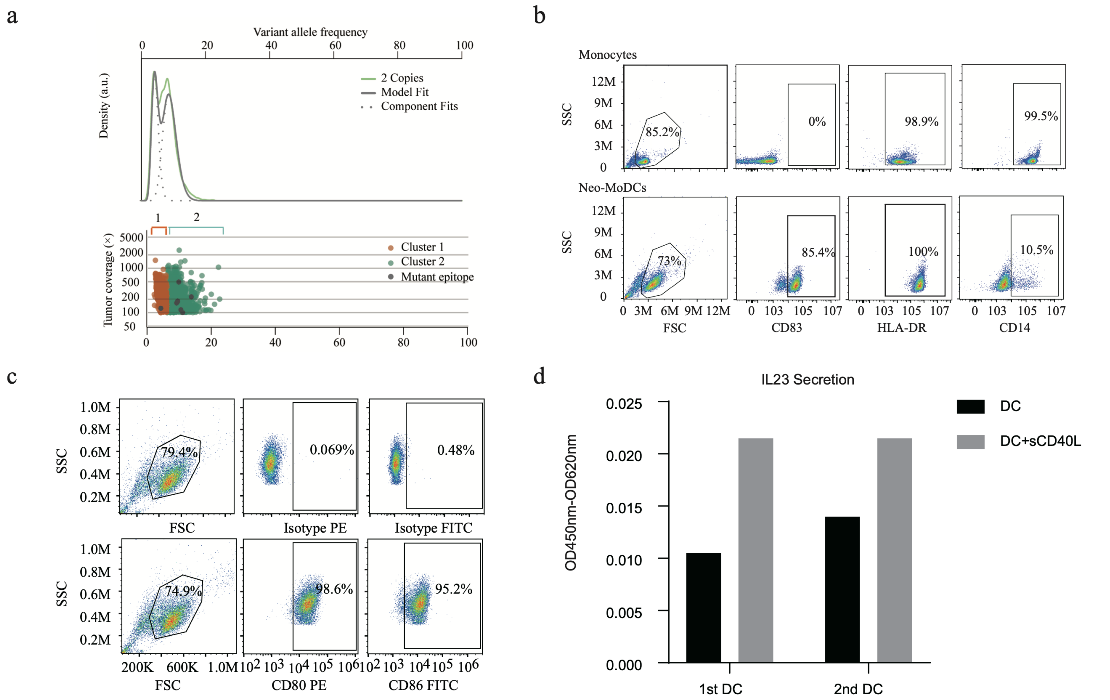


**Supplementary Figure 2. Target of Neo-MoDC and Neo-MoDC vaccine characterization. a** The clonality of mutant epitopes. The upper panel, Kernel density plots of variant allele frequency across regions; the lower panel, the distribution of mutant epitopes in mutation clusters. **b** Maturation of the Neo-MoDC was assessed by HLA-DR, CD83, and CD14 staining. Monocytes were served as the control. We can see that mature Neo-MoDCs upregulated CD83 expression, and downregulated CD14. SSC, side scattering; FSC, forward scattering. **c** Maturation of the Neo-MoDC was assessed by CD80 and CD86 staining. d. IL23 secretion from Neo-MoDC cells during maturation (PMID: 31029042) was measured. DC were stimulated 24h with 250ng/mL recombinant sCD40L (Pepro Tech, AF-310-02), and the IL-23 concentration in the supernatants were determined using Human IL-23 Precoated ELISA kit (Dakewei). We produced two batches (1st DC and 2nd DC) of Neo-MoDCs using the same protocol to make sure we had enough MoDCs for vaccination.

| ASP pool 1 | | |
|---|---|---|
| **TMEM38B F251V** | | DTLSWMLFGWQQPVSSCEKKSEAKSPS |
| | 1 | DTLSWMLFGWQQPVS |
| | 2 | WMLFGWQQPVSSCEK |
| | 3 | GWQQPVSSCEKKSEA |
| | 4 | PVSSCEKKSEAKSPS |
| **TIGD3 V118M** | | KAKELADIMGQDFMPSIGWLVRWKRRN |
| | 1 | KAKELADIMGQDFMP |
| | 2 | LADIMGQDFMPSIGW |
| | 3 | MGQDFMPSIGWLVRW |
| | 4 | FMPSIGWLVRWKRRN |
| **ZZEF1 R898H** | | TEQEHKQSLQLTFHSLCTYFSDKDPGG |
| | 1 | TEQEHKQSLQLTFHS |
| | 2 | HKQSLQLTFHSLCTY |
| | 3 | LQLTFHSLCTYFSDK |
| | 4 | FHSLCTYFSDKDPGG |
| **URB1 R421W** | | SRAFQTREFIPLPWLLAMVMVTTVPLV |
| | 1 | SRAFQTREFIPLPWL |
| | 2 | QTREFIPLPWLLAMV |
| | 3 | FIPLPWLLAMVMVTT |
| | 4 | PWLLAMVMVTTVPLV |

| ASP pool 2 | | |
|---|---|---|
| **DLEC1 R331C** | | ARMESRNHFLKNPCFFPPNTRYGGKSL |
| | 1 | ARMESRNHFLKNPCF |
| | 2 | SRNHFLKNPCFFPPN |
| | 3 | FLKNPCFFPPNTRYG |
| | 4 | PCFFPPNTRYGGKSL |
| **TMTC1 C313Y** | | VAERVLYMPSMGYYILFVHGLSKLCTW |
| | 1 | VAERVLYMPSMGYYI |
| | 2 | VLYMPSMGYYILFVH |
| | 3 | PSMGYYILFVHGLSK |
| | 4 | YYILFVHGLSKLCTW |
| **MTA2 R92W** | | QRHQLKHRELFLSWQFESLPATHIRGK |
| | 1 | QRHQLKHRELFLSWQ |
| | 2 | LKHRELFLSWQFESL |
| | 3 | ELFLSWQFESLPATH |
| | 4 | SWQFESLPATHIRGK |
| **TDP1 K112Rfs.101** | | TLEPSTSRIFYLLYLGRLFLQLSLTTA |
| | 1 | TLEPSTSRIFYLLYL |
| | 2 | STSRIFYLLYLGRLF |
| | 3 | IFYLLYLGRLFLQLS |
| | 4 | LYLGRLFLQLSLTTA |

**Supplementary Figure 3. Mutant assay peptide (APS) pools construction.** Red letters denote mutations.

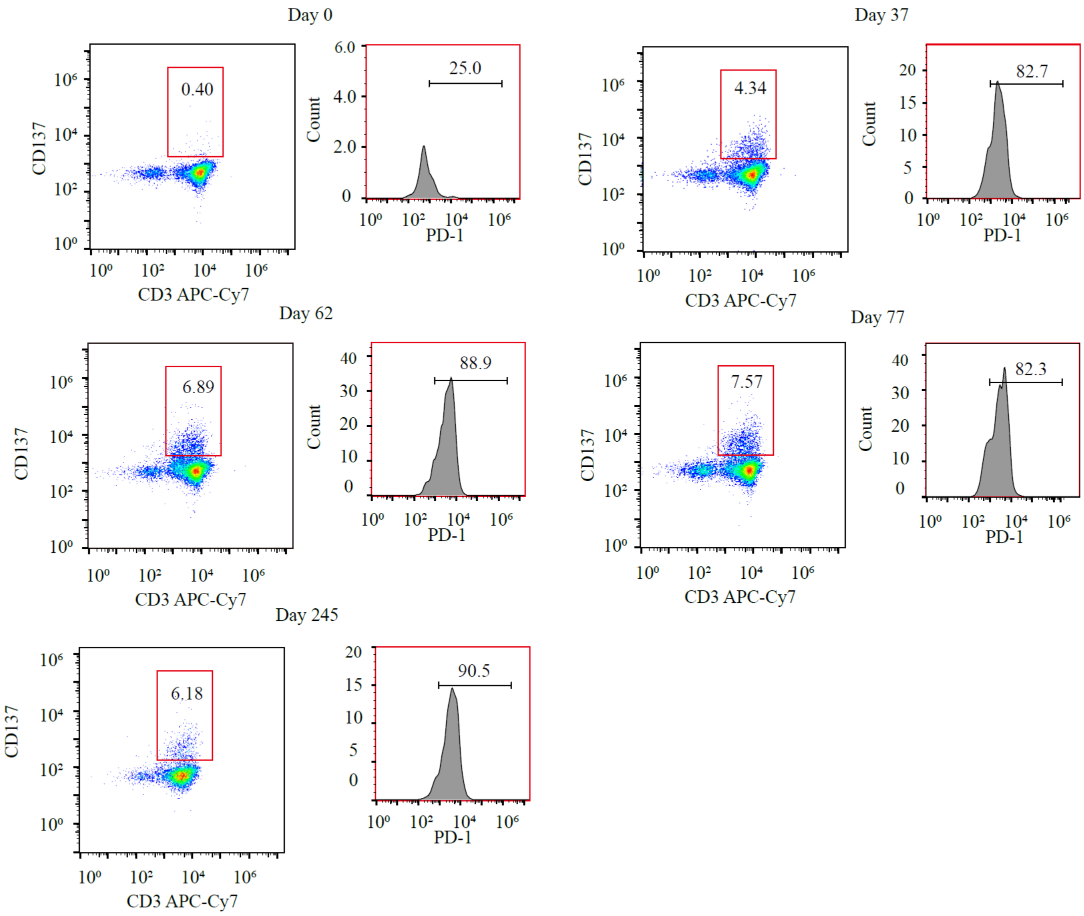


**Supplementary Figure 4.** Increased PD-1 level on activated T cell surface after mutant-peptides stimulation. PBMCs from the patient were collected at different time points during the treatment and expanded *in vitro* for 10 days in the presence of mutant peptides. After resting in cytokine-free culture medium overnight, the cells were re-stimulated with mutant peptides for 20 h. Then the PBMCs were co-stained with CD137-PE (BD Biosciences, clone:4B4-1, 555956), CD3-APC-Cy7(BD Biosciences, clone: SK7, 557832), PD-1(CD279)-APC (BD Biosciences, clone: MIH4, 558694) analyzed by FACS. The use of each antibody was 5 µl per million cells in 100 µl staining volume. Results showed that Neo-MoDC vaccination alone (day37, 62) was able to activate T cells. And T cells after initial vaccination express higher level of PD-1 on their surface.

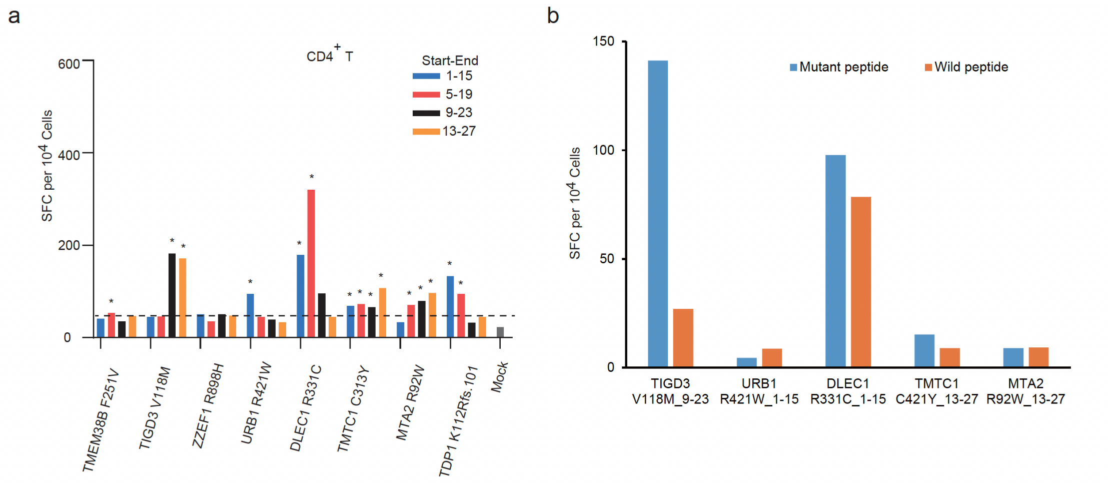


**Supplementary Figure 5. CD4$^+$ T cells response to 15mer peptides. a** Mapping of CD4$^+$ T cells response to each 15mer peptide. Each 27mer mutant peptide was cutting into four 15mer peptides (denoted by blue, red, black, and orange bars) whose start and end positions in the 27mer peptide are 1-to-15, 5-to-19, 9-to-23, and 13-to-27. Dotted line, 2 folds of mock; *, SFC numbers of mutant epitopes were more than twice as much as that in the mock. Data shown here were the mean value of two replicates. **b** T cells response to mutant peptides and their corresponding widetype peptides.

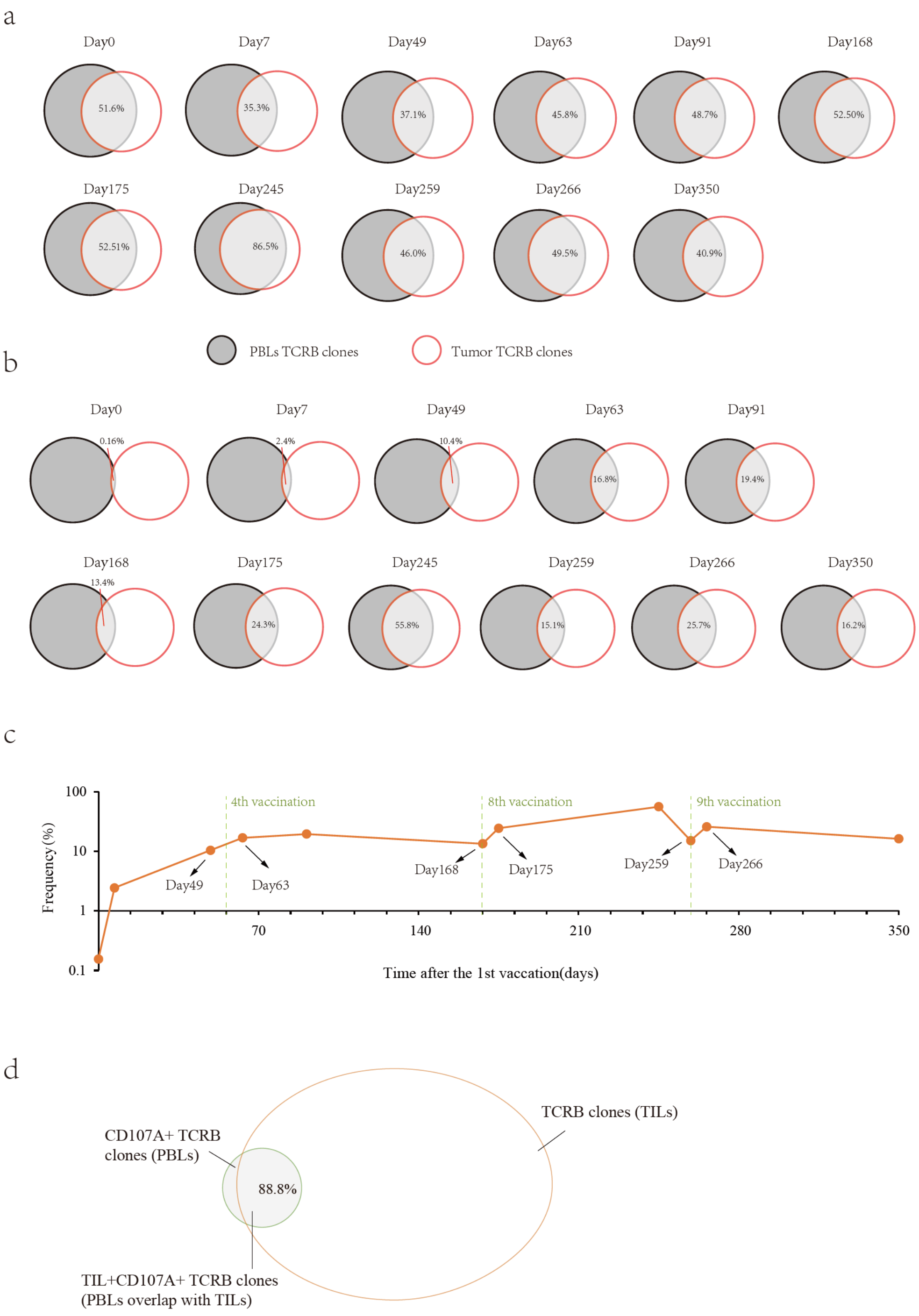


**Supplementary Figure 6. TCRB clones overlap at each PBL sampling time point between tumor tissue and blood.** **a** Venn diagrams showed that a large fraction of TCRB clones in PBLs could be found in the tumor tissue. **b** Overlap of tumor enriched TCRB clones (with frequency in primary tumor tissue 10-fold higher than that in day0 PBLs), and their percentage in PBLs. **c** The percentage of tumor enriched TCRB clones in PBLs at each PBLs sampling time point. **d** Shared TCRB clones between mutant peptide activated T cells in PBLs (CD107A$^{+}$) and T cells in tumors.

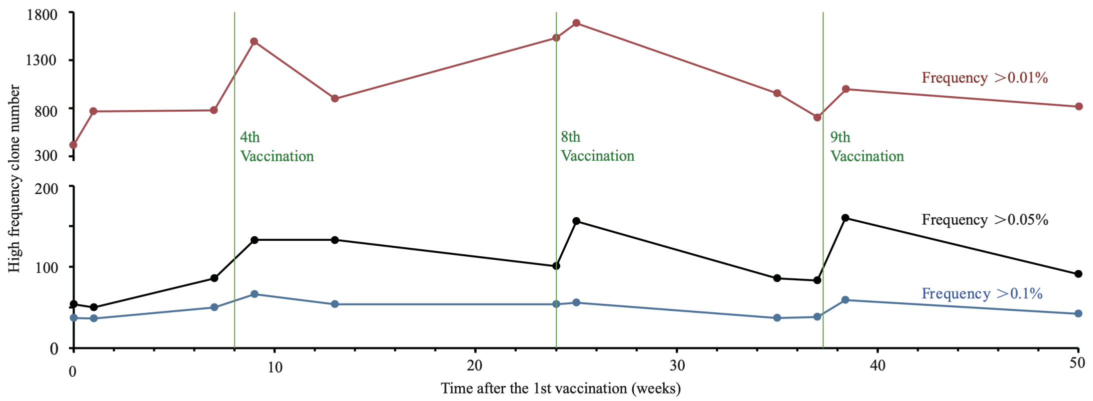


**Supplementary Figure 7. The number of high-frequency clones during treatment.**
